# A Novel Label-Free Optical Biosensor Using Synthetic Oligonucleotides from *E. coli* O157:H7: Elementary Sensitivity Tests

**DOI:** 10.3390/s90604890

**Published:** 2009-06-19

**Authors:** Zehra Banu Bahşi, Aligül Büyükaksoy, Sinan Mert Ölmezcan, Fethi Şimşek, Muhammed Hasan Aslan, Ahmet Yavuz Oral

**Affiliations:** 1 Dept of Environmental Engineering, Gebze Institute of Technology, Kocaeli, Turkey; E-Mail: bbahsi@gyte.edu.tr; 2 Dept of Materials Science and Engineering, Gebze Institute of Technology, Kocaeli, Turkey; E-Mails: aligul@gyte.edu.tr (A.B.); mertolmezcan@gmail.com (S.M.O.); 3 Nanotechnology Laboratory, Doga Nanobiotech, Istanbul, Turkey; E-Mail: fsimsek@dogananobiotech.net; 4 Dept of Physics, Gebze Institute of Technology, Kocaeli, Turkey; E-Mail: maslan@gyte.edu.tr

**Keywords:** sol-gel, optical DNA biosensor, prism coupler

## Abstract

SiO_2_-TiO_2_ thin films for use as fiber optic guiding layers of optical DNA biosensors were fabricated by the sol-gel dip coating technique. The chemical structure and the surface morphology of the films were characterized before immobilization. Single probe DNA strands were immobilized on the surface and the porosity of the films before the hybridization process was measured. Refractive index values of the films were measured using a Metricon 2010 prism coupler. On the surface of each film, 12 different spots were taken for measurement and calculation of the mean refractive index values with their standard deviations. The increased refractive index values after the immobilization of single DNA strands indicated that immobilization was successfully achieved. A further refractive index increase after the hybridization with target single DNA strands showed the possibility of detection of the *E. coli* O157:H7 EDL933 species using strands of 20-mers (5′-TAATATCGGTTGCGGAGGTG -3′) sequence.

## 1. Introduction

Biosensors are widely used in environmental monitoring due to their capability for accurate real time detection of low levels of chemical and biological contaminants. In addition, they have the advantage of being cost effective, which is very significant in environmental control [1,2].

DNA biosensors are composed of a solid surface (transducer), a single strand DNA immobilized onto the surface (probe) and a sequence specific single strand DNA (target) whose existence or amount is tested in the sample. The main principle of DNA biosensors is simply based on the detection of hybridization of these DNA sequences. The methods for detection can be acoustic, electrochemical, or optical [3]. Optical biosensors are widely used due to their ability to give fast results with high signal/noise ratios. In addition, they can be used *in situ* and are minimally invasive for *in vivo* measurements. Optical biosensing can be direct or indirect. Indirect measurements require the labeling of the analytes which can cause alterations in their biochemical activity. Thus, direct (label-free) optical biosensors are preferred [4].

A novel approach to label free optical biosensing is the use of a prism coupler as the transducer. A prism coupler is a device that determines the refractive index/birefringence and the thickness of a dielectric film with high precision [5]. Thus, it is well-suited for the measurement of minute changes in the refractive index caused by the immobilization of nano-sized biomolecules (DNA, enzyme etc.) on the dielectric film without labeling [6].

The sol-gel technology is being increasingly used for the development of biosensors due to its simplicity and versatility [7]. It is a convenient way to produce transparent, amorphous and homogeneous dielectric thin films on various substrates. Therefore, it is an ideal technique for the deposition of the optical waveguide layers which can be tested in prism couplers. In addition, sol-gel technology makes it possible to fabricate multi-component oxide thin films with perfect chemical composition control and precise tailoring of the refractive index of the thin films [8,9].

Waterborne pathogens (*Escherichia coli, Salmonella*, etc.) cause numerous fatal and non-fatal infections [10]. The presence of “coliforms” which include four genera (*Escherichia, Citrobacter Enterobacter* and *Klebsiella*) is normally used as an indicator to monitor for potential enteric pathogen contamination of waters [11]. *Escherichia coli (E. coli)* is chosen as an indicator of fecal contamination in surface waters in preference to the heterogeneous fecal coliform group [12]. One of hundreds of strains of the bacterium Escherichia coli is E. coli O157:H7 which is the emerging cause of many foodborne and waterborne illnesses [13]. Water resources contaminated with feces such as surface water runoff, irrigation water, insufficiently chlorinated municipal water, and swimming water (pools, lakes, beaches) can contain *E. coli* O157:H7 [14].

Several methods are used for the detection of *E. coli* in water, including microbiological, serological, and immunological procedures [11]. In this study, we have developed a novel sol-gel derived SiO_2_-TiO_2_ thin film based optical DNA biosensor using a prism coupler. The DNA probe targeting the 20-mers (5′-TAATATCGGTTGCGGAGGTG -3′) has been designed to detect the *E. coli* O157:H7 EDL933 species.

## 2. Experimental

### 2.1. SiO_2_-TiO_2_ Thin Films Preparation

**Figure 1.**
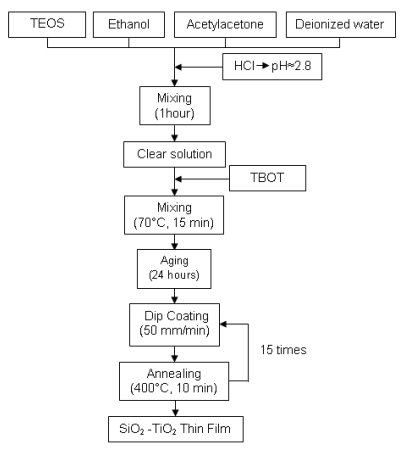
Flowchart of SiO_2_–TiO_2_ thin film fabrication.

The solution was prepared by using tetraethyl orthosilicate (TEOS, C_8_H_20_O_4_Si) and tetra-*n*-butyl orthotitanate (TBOT, C_16_H_36_O_4_Ti) as SiO_2_ and TiO_2_ sources, respectively. First, TEOS was mixed with ethanol (C_2_H_5_OH), acetylacetone (C_5_H_8_O_2_) and deionized water (H_2_O). The molar ratio of mixture (TEOS: ethanol: acetylacetone: deionized water) was 1:0.5:1:3. Next, hydrochloric acid (HCl) was added dropwise as a catalyst. The measured values of pH and viscosity of the solution were 2.8 and 1.26 mPa.s at 25 °C, respectively. After mixing for one hour, a clear solution was obtained. Then TBOT was added to the solution until the TEOS : TBOT ratio was 1:1. The final solution was stirred at 70 °C for 15 minutes. After aging for 24 hours, the solution was deposited on glass substrates by dip coating with a withdrawal speed of 50 mm/min. Following each coated layer, the films were dried at 400 °C for 10 minutes. The SiO_2_-TiO_2_ thin films had sufficient thickness after 15 layers of coating (Figure 1). The chemical structure of the films was examined by Fourier Transformed Infrared Spectroscopy (FTIR, Bio–Rad Tropical Option for 175 C) and the surface morphology of the films was observed by Scanning Electron Microscopy (SEM, Philips XL 30 SFEG).

### 2.2. DNA Immobilization & Hybridization

The surface of the SiO_2_-TiO_2_ thin films were modified before DNA immobilization. First, the films were silanized with 2% 3-(triethoxysilyl)propylamine (APTES, C_9_H_23_NO_3_Si) in (1:1) deionized water and methanol (CH_3_OH) mixture for 20 minutes. After rinsing with deionized water, the films were dried at 100 °C for 10 minutes. Next, the surface of the films were modified with 0.2% 1,4-phenylene diisothiocyanate (PDC, C_6_H_4_(NCS)_2_) in dimethyl sulfoxide [DMSO, (CH_3_)_2_ SO] for 2 hours to form a crosslinker monolayer atop the APTES monolayer. Then the film was rinsed with methanol and acetone (CH_3_COCH_3_), respectively, and dried with nitrogen. Before getting ready for DNA immobilization, the surfaces of the films were treated with 20 mM 4-(2-hydroxyethyl)-1-piperazineethanesulfonic acid (HEPES, C_8_H_18_N_2_O_4_S) buffer for 10 minutes. After rinsing with HEPES buffer, the surface was dried with nitrogen. Amine modified synthetic probe and target oligonucleotides were purchased from The Midland Certified Reagent Company (Texas, USA). The amine modified DNA probe which was specific to *Escherichia coli* O157:H7 of GeneID: 957271, was dissolved in HEPES buffer to obtain a 100 μM solution. Afterwards, the films were exposed to this solution containing the probe DNA oligonucleotide of sequence 5′-(C6Amino) CACCTCCGCAACCGATATTA-3′ in an incubator at 37 °C for 1 hour. The probe DNA immobilization was completed after rinsing with HEPES buffer and drying with nitrogen.

**Figure 2.**
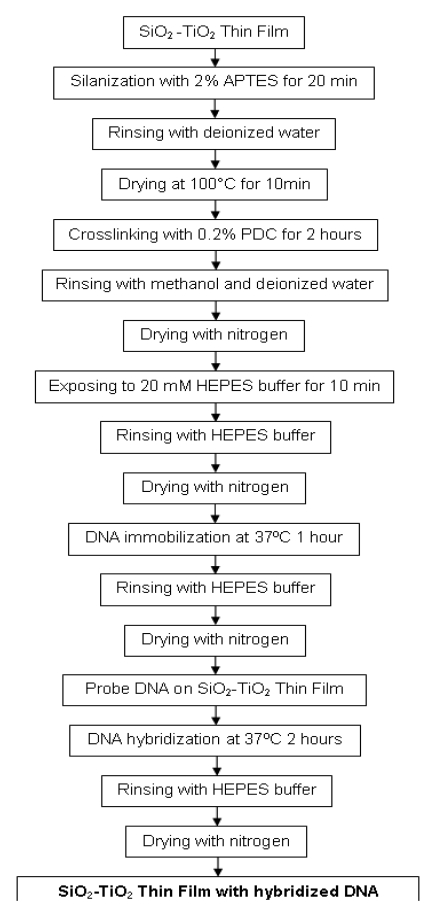
The flowchart of DNA immobilization and hybridization process.

For hybridization, the target DNA oligonucleotide (5′-TAATATCGGTTGCGGAGGTG-3′) of concentration 100 μM was applied on the probe immobilized films at 37 °C for 2 hours. Then hybridized films were rinsed with HEPES buffer and dried with nitrogen (Figure 2). Prism coupler measurements were carried out before the probe DNA immobilization, after the probe DNA immobilization, and after the hybridization processes to determine the refractive index changes after each process.

### 2.3. Prism Coupler Setup

**Figure 3.**
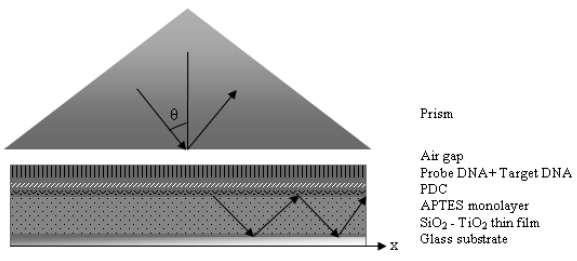
Schematic view of the experimental setup.

The films are brought into contact with the base of a prism until there is an air gap in the nanometer level between the film and the prism. An incident laser beam hits the base of the prism and total internal reflection occurs due to the higher refractive index of the prism (*np*) when compared to air. The reflected laser beam strikes to a photodetector and the light intensity is measured. At certain angles of incidence, *θ*, tunneling of photons takes place. The tunneling photons go through the air gap and enter the dielectric film, which causes an instant drop in the intensity of light reaching the detector [5]. For this tunneling to occur properly, the air gap between the prism and the film should be smaller than the wavelength of the incident beam. The angle θ determines the phase velocity of the incident wave in the prism and in the gap, along the surface of the films, υ^(^*^i^*^)^ = (c/n_p_) sin θ. The strong coupling of the light only occurs when *θ* is chosen such that υ^(^*^i^*^)^ equals to the phase velocity of one of the characteristic modes [15] (Figure 3). These angles are called mode angles.

## 3. Results and Discussion

**Figure 4.**
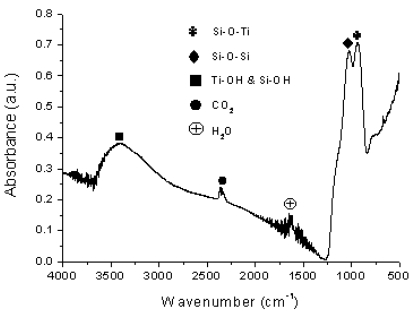
Fourier Transformed Infrared Spectroscopy (FTIR) graph of SiO_2_–TiO_2_ thin films.

The chemical structure of SiO_2_-TiO_2_ hybrid coatings was investigated by FTIR measurements. The peak occurring around 940 cm^-1^ corresponds to the vibration of Si–O–Ti bonds and the peak at around 1,030 cm^-1^ is attributed to the characteristic vibration of Si–O–Si bonds [16,17]. The broad band around 3,400 cm^-1^ arises from the vibration of Ti–OH and Si–OH groups in the film [16,18]. The presence of atmospheric CO_2_ and adsorbed water were revealed by the peaks occurring at around 2,350 cm^-1^ and 1,620 cm^-1^ respectively (Figure 4).

The hybrid thin films were fabricated to provide a layer with a higher refractive index than the glass substrate for waveguiding properties and to obtain a surface with available sites for the self assembly of APTES. The presence of Ti–OH and Si–OH groups in sol-gel derived films, confirmed by FTIR measurement, are suitable sites for the binding of the Si atoms of APTES. The bifunctional crosslinker PDC contains carbon atoms which favor covalent bond formation with nitrogen at both ends. Thus, due to the presence of nitrogen atoms in the amino groups of amino modified DNA and APTES, crosslinking via C–N bonds take place.

**Figure 5.**
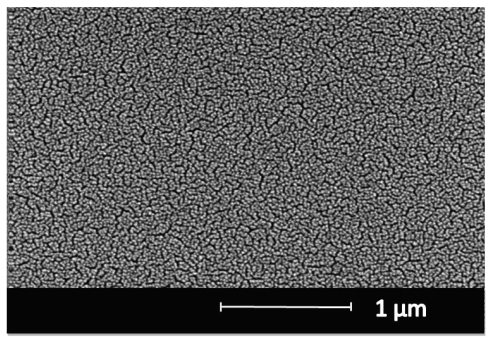
Scanning Electron Microscopy imaging of SiO_2_–TiO_2_ thin films.

The SEM micrograph of the hybrid coatings showed a homogeneous surface morphology with uniformly distributed nano-sized pores. The high homogeneity and high surface area provides a suitable surface for uniform DNA binding and consequently makes the prism coupler measurements reliable. It is also clear that the film had an amorphous structure which favors the waveguiding ability of the film due to lack of grain boundaries scattering the transient light (Figure 5). Even though in our configuration, the laser is not traveling through the films, waveguiding property is still required. This is due to significant decrease of FWHM of the peaks in waveguide structures due to low loss of light to the lower index substrate. Sensitivity of the proposed sensor is strongly dependent on the FWHM of the peaks obtained. The calculated FWHM values were around 0.1° suggesting good sensitivity for the sensor.

Metricon 2010 Prism coupler measurements were performed before the probe DNA immobilization, after the probe DNA immobilization, and after the hybridization processes. To increase accuracy, the measurements were taken from 12 different spots and averaged. The theoretical refractive index of SiO_2_-TiO_2_ hybrid material is estimated as 2.01 by calculating weighted average of the refractive indices of SiO_2_ (∼1.46) and amorphous TiO_2_ (∼ 2.57). The measured mean refractive index value before the probe DNA immobilization was 1.6928 which is significantly lower than the theoretically calculated refractive index. This is due to the porous structure of the films as can be clearly seen in the SEM micrograph. From the measured refractive index value, volume percent of porosity was estimated to be around 32%.

**Figure 6.**
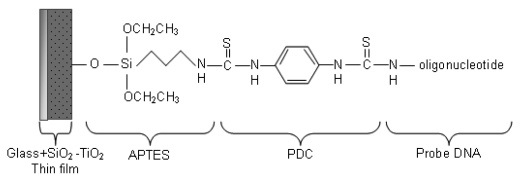
The proposed surface structure after probe DNA immobilization.

**Figure 7.**
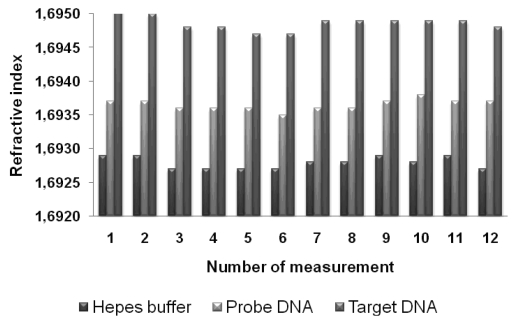
The bar chart of refractive index values versus number of measurements.

The proposed surface structure after the immobilization is presented in Figure 6. A similar surface chemistry was also suggested by Festag *et al.* on glass substrates [19]. The mean refractive index value of the film after the probe DNA immobilization was 1.6937. From the increase, it can be concluded that in addition to the probe DNA strands immobilized on the surface, there were also strands immobilized inside the nanosized pores. Since DNA has a refractive index (∼ 1.5) higher than the air inside the pores (∼ 1.0), the slight increase in the refractive index is considered to be result of DNA strands inside the pores. The mean refractive index value after the target DNA hybridization was 1.6949. Further increase of refractive index can be explained by the fact that the amount of higher refractive index material (DNA strands) inside the pores after the hybridization process. The refractive index versus number of measurements graph shows the increase of refractive indices at each step (Figure 7), while Table 1 displays the mean of refractive index values and their standard deviations.

**Figure 8.**
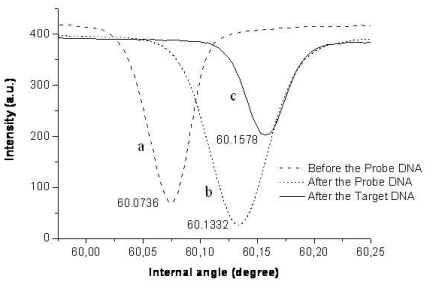
The resonant angles: (a) before probe DNA immobilization; (b) after probe DNA immobilization; (c) after target DNA hybridization processes.

Figure 8 shows the angle of resonance after each step. The measured resonant angles were 60.0736° before immobilization, 60.1332° after immobilization and 60.1578° after hybridization. The increase of refractive index and resonant angle is in agreement with the findings of Rong *et al.* [20,21] who have also employed prism coupler for DNA detection but used porous silicon as the waveguide. They have reported an achieved detection limit of 2.17 μM. The detection limit obtained by our results was in the same range (8.13 μM) and corresponded to a resonance shift of 0.002° (the resolution of prism coupler). The different waveguide materials and different porosity levels used in current work may have resulted in a slightly lower probe density and a higher detection limit. Nevertheless, our preliminary results suggest that sol–gel derived SiO_2_-TiO_2_ thin films are well-suited for specific oligonucleotide detection by a prism coupler especially after further optimization of the probe density.

## 4. Conclusions

Homogeneous SiO_2_-TiO_2_ hybrid films were produced by a sol-gel dip-coating method to provide a thin layer with a refractive index higher than the glass substrate for prism coupler measurements. In addition, the films contained available sites (nanopores) for the self assembly of APTES. Probe oligonucleotides were immobilized on the thin films via APTES and cross-linking PDC monolayers. Target DNA strands were effectively hybridized with probe DNA strands. The immobilization and hybridization processes were confirmed by the increasing refractive index values. All refractive index measurements were performed by using Metricon 2010 prism coupler to accomplish a novel label free optical biosensor. Our preliminary results suggested that instead of conventional detection methods, specific sequences of DNA of coliforms like *E. coli* O157:H7 EDL933 species have the possibility to be rapidly detected from the refractive index changes caused by hybridization using Metricon 2010 prism coupler.

## Figures and Tables

**Table 1.** Mean refractive index values and their standard deviations.

	**HEPES Buffer**	**Probe DNA**	**Target DNA**
**Mean refractive index values**	1.6928	1.6937	1.6949
**Standard deviation**	0.00009	0.00008	0.00012
